# Nanomedicine strategies for remodeling the solid tumor microenvironment: stromal targeting, hypoxia modulation, and photodynamic immunotherapy

**DOI:** 10.3389/fonc.2026.1914117

**Published:** 2026-08-27

**Authors:** Mohd Ahmar Rauf, Duy Luong, Farhan Shah, Arun K. Iyer

**Affiliations:** 1 Department of Internal Medicine, Heme/Oncology Unit, University of Michigan, Ann Arbor, MI, United States; 2 Department of Pharmaceutical Sciences, Eugene Applebaum College of Pharmacy and Health Sciences, Wayne State University, Detroit, MI, United States; 3 Department of Medical and Translational Biology, Umea University, Umea, Sweden; 4 Molecular Imaging Program, Karmanos Cancer Institute, Wayne State School of Medicine, Detroit, MI, United States

**Keywords:** cancer-associated fibroblasts, drug delivery, hypoxia, immunotherapy, mRNA vaccines, nanomedicine, pancreatic cancer, photodynamic therapy

## Abstract

The tumor microenvironment is more than a backdrop for malignant cells: stromal fibroblasts, extracellular matrix, abnormal vasculature, hypoxia, and immunosuppressive myeloid and lymphoid populations act together to limit both drug delivery and treatment response. Here, we examine nanomedicine along three linked axes: stromal remodeling, hypoxia control, and photodynamic immune activation. CAF-directed and matrix-responsive carriers can improve perfusion and intratumoral transport; however, because fibroblasts are heterogeneous, subtype-selective reprogramming is safer than wholesale depletion, which can worsen outcomes. Oxygen-generating, hypoxia-activated, and oxygen-conserving Type I photochemical systems address both pre-existing hypoxia and the additional oxygen depletion that PDT itself causes. We consider immune-active nanocarriers, such as STING agonists and mRNA vaccines, because stromal access and hypoxic stress shape their distribution, while PDT-induced immunogenic cell death supplies the antigenic and inflammatory signals on which these platforms are built. Pancreatic ductal adenocarcinoma remains the archetype of desmoplasia, yet we deliberately contrast it with glioblastoma, breast, ovarian, and other solid tumors, whose microenvironments differ in important ways. Translation is still held back by variable and often low tumor delivery, protein-corona formation, off-target clearance, manufacturing complexity, and inconsistent patient selection. The more realistic path forward is not ever-greater carrier complexity, but biomarker-guided, mechanism-matched platforms with measurable microenvironmental endpoints and a clear route to scalable production.

## 1 Introduction

Solid tumors present problems that extend beyond the malignant cells themselves. Despite real progress in mapping oncogenic signaling, building targeted therapies, and advancing immunotherapy, many patients with advanced diseases, such as pancreatic ductal adenocarcinoma, triple-negative breast cancer, and glioblastoma, still show only limited or short-lived responses. This gap between deep biological understanding and durable clinical benefit points to a recurring blind spot in conventional therapeutic design: the tumor microenvironment is treated as background rather than as a central driver of resistance (1–3). It is neither passive nor inert in nature. Cancer cells evolve alongside stromal, vascular, metabolic, and immune components to create a protective niche. A dense desmoplastic stroma rich in cancer-associated fibroblasts and collagenous extracellular matrix increases interstitial fluid pressure and erects physical barriers that prevent small-molecule drugs and nanocarriers from penetrating the tumor. Abnormal, poorly perfused vessels create steep oxygen gradients, and the resulting hypoxic regions drive adaptive resistance and metabolic reprogramming in tumors. In addition to these physical and metabolic constraints, a strongly immunosuppressive environment exists in which regulatory T-cells, tumor-associated macrophages, and myeloid-derived suppressor cells cooperate to blunt antitumor immunity. Together, stromal rigidity, hypoxia, and immune suppression shield the tumor from treatment while facilitating its progression and relapse (4–7). This implies a shift from tumor-centric to microenvironment-centric strategies. Nanomedicine fits this shift well: it can carry drugs deep into tumor tissue and, at the same time, remodel the microenvironment itself. Nanoparticles can be engineered to normalize stromal architecture, reprogram immunosuppressive populations, deliver or generate oxygen in hypoxic zones, and release their payloads in response to tumor-specific cues (8–10). In this review, we use pancreatic ductal adenocarcinoma as a prototypical desmoplastic tumor, as its large cancer-associated fibroblast compartment, hyaluronan- and collagen-rich matrix, vascular compression, and severe hypoxia create several delivery barriers simultaneously. However, it is not a universal surrogate. Anatomical access, stromal composition, vascular permeability, immune architecture, and routes of spread differ markedly across solid tumors; therefore, carrier size, route, target, and release logic must be tailored to each disease rather than extrapolated wholesale from pancreatic cancer (1–3, 8, 11). Recent work on cancer-associated fibroblasts supports this notion: stromal targets should be matched to the disease context rather than to a single assumed fibroblast program (12).

Photodynamic therapy (PDT) presents significant design challenges in tumor microenvironment (TME)-directed nanomedicine. When activated by light, photosensitizers produce reactive oxygen species (ROS) that can harm tumor cells and blood vessels. Additionally, they can trigger immunogenic cell death, which is marked by calreticulin exposure, the release of ATP and HMGB1, and enhanced presentation of tumor antigens. However, the potential of this therapy is constrained by microenvironmental barriers that also hinder many treatments for solid tumors. Conventional Type II PDT relies on molecular oxygen, which can be further depleted during the therapy, leading to hypoxia and possibly activating adaptive resistance pathways such as HIF, PD-L1, STAT3, and IDO signaling. In this context, nanocarriers are most effective when they do more than merely deliver a photosensitizer. Their true value lies in connecting PDT with improved access to the stroma, replenishing oxygen or utilizing oxygen-sparing photochemistry, and enabling spatially controlled immune activation. This approach allows PDT to serve as part of a comprehensive strategy for remodeling the microenvironment rather than functioning as a standalone cytotoxic treatment. Consequently, the review is structured around three interconnected strategies: overcoming stromal barriers through CAF targeting and ECM normalization, remodeling hypoxic niches using oxygen-generating, hypoxia-triggered, and oxygen-sparing systems, and reprogramming immune suppression via nanoparticle-enabled innate and adaptive immunotherapy. We will also address clinical translation, multi-cargo delivery, and the integration of PDT within these frameworks (13–17). This review is unique in that it considers stromal remodeling, hypoxia control, and photoimmunotherapy as one interdependent design problem rather than three separate topics. Where broad pan-cancer nanotechnology surveys catalog platforms (18), we ask how CAF state, oxygen availability, myeloid suppression, light delivery, and cargo sequence jointly determine performance—and we tie those mechanisms to clinical translation that has both failed and succeeded.

## 2 Stromal targeting: reprogramming the tumor microenvironment

**Figure 1:**
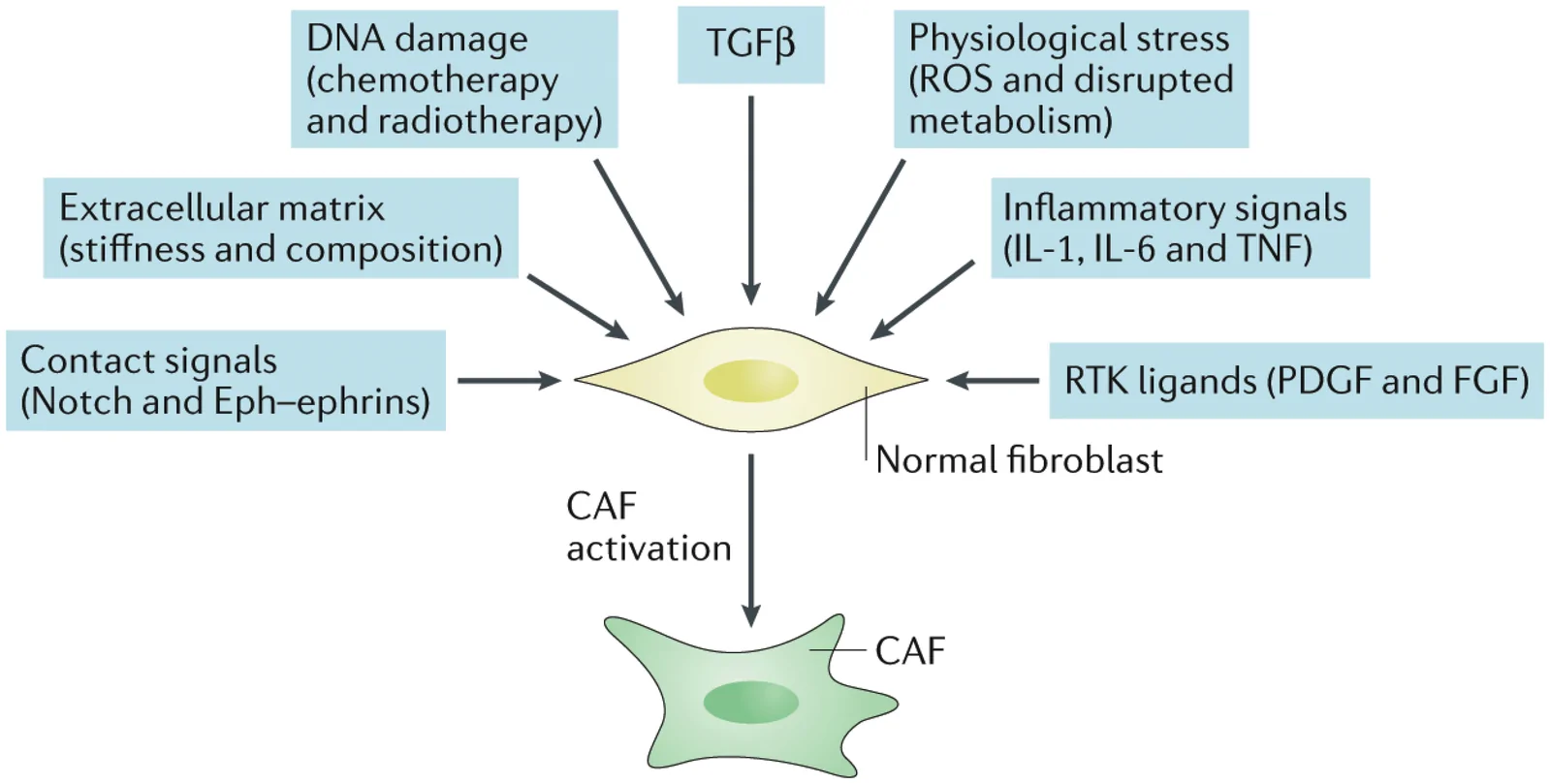
Fibroblast activation signals in the tumor microenvironment. Multiple cues, including TGF-β, DNA damage from therapy, inflammatory cytokines (IL-1, IL-6, TNF), RTK ligands (PDGF, FGF), and ECM stiffness, drive the trans differentiation of normal fibroblasts into cancer-associated fibroblasts. Adapted from Sahai et al. (8).

The tumor microenvironment (TME) is a complex ecosystem in which malignant cells, stromal components, immune infiltrates, and elements of the extracellular matrix (ECM) coexist and engage in crosstalk that influences tumor progression, metastasis, and therapeutic resistance. Among the stromal constituents, cancer-associated fibroblasts (CAFs) are the most prevalent cell type in numerous solid tumors. While normal fibroblasts contribute to maintaining tissue homeostasis, CAFs exhibit a persistently activated phenotype characterized by increased proliferation, altered secretory programs, and extensive ECM remodeling. Through reciprocal signaling with tumor cells, these activated fibroblasts contribute to the formation of a pro-tumorigenic niche, which presents significant barriers to drug delivery and immune surveillance in the TME. Consequently, understanding CAF heterogeneity and functional plasticity is crucial for designing nanomedicine platforms that can penetrate the stromal barrier and reach malignant cells (11, 12). CAFs originate from various cellular sources, including resident tissue fibroblasts, bone marrow-derived mesenchymal stem cells (MSCs), pericytes, and endothelial cells undergoing endothelial-to-mesenchymal transition (End). This diverse provenance directly contributes to the notable heterogeneity observed within CAF compartments. Transcriptional profiling has identified several distinct CAF subsets, including myofibroblastic CAFs (myCAFs) and inflammatory CAFs (iCAFs), each with specific functional specializations. MyCAFs, characterized by high expression of alpha-smooth muscle actin (αSMA) and a contractile phenotype, are primarily responsible for ECM deposition and structural remodeling. In contrast, iCAFs secrete elevated levels of pro-inflammatory cytokines, including interleukin-6 (IL-6), interleukin-8 (IL-8), and chemokine ligand 2 (CCL2), which help sustain an immunosuppressive environment and weaken anti-tumor immunity. Recently, a third subset, antigen-presenting CAFs (apCAFs), has been identified. These cells express major histocompatibility complex class II (MHC-II) and may perform immunomodulatory functions. This phenotypic diversity underscores the challenge of developing therapies that selectively target pathogenic CAF subsets while preserving the fibroblasts essential for normal tissue functions (13–15). The activated CAF state is maintained by persistent stimulation from tumor-derived growth factors, most notably transforming growth factor-beta (TGF-β). Through Smad2/3 signaling, TGF-β drives the transdifferentiation of quiescent fibroblasts into myofibroblastic CAFs and induces the expression of α-smooth muscle actin (α-SMA), vimentin, and fibronectin. Simultaneously, sonic hedgehog (SHH) signaling from tumor cells activates GLI transcription factors in stromal fibroblasts, promoting a robust desmoplastic response to tumor growth. The resulting collagen-rich stroma does more than create a physical blockade; it raises interstitial fluid pressure (IFP), compresses tumor vasculature, worsens hypoxia, and limits the extravasation of nanoparticles. In parallel, CAF-secreted TGF-β directly suppresses T-cell function and promotes regulatory T-cell (Treg) differentiation, reinforcing an environment that is deeply hostile to immunity (16, 17). Several molecular targets have been identified for selective CAF-directed nanomedicine. Fibroblast activation protein-alpha (FAPα), a type II transmembrane serine protease with dipeptidyl peptidase and endopeptidase activity, is highly expressed in CAFs in multiple tumor types but is largely absent in normal adult tissues. This restricted expression pattern, combined with its functional role in tumor progression, makes FAPα an attractive target for therapeutic inhibition and targeted drug delivery. Preclinical studies using FAPα-targeted nanoparticles have demonstrated improved tumor penetration and superior therapeutic outcomes, particularly when combined with chemotherapy and immunotherapy (18, 19). Alpha-smooth muscle actin (αSMA) is another important marker, especially enriched in the myCAF subset, which generates contractile forces behind stromal compaction. Platelet-derived growth factor receptor-alpha (PDGFRα) and discoidin domain receptor 2 (DDR2), both of which mediate fibroblast responses to collagen signaling, have emerged as viable targets for CAF-directed therapy. Tenascin-C, an ECM glycoprotein abundantly expressed in the tumor stroma, serves as a useful epitope for nanoparticle homing. The molecular targets and signaling pathways underlying CAF-directed nanomedicine are shown in **Figure 1** (20).

Subtype-aware design is important because myCAF, iCAF, and antigen-presenting CAF states are plastic and spatially organized, not fixed lineages. A myCAF-rich matrix may require approaches that reduce contractility or collagen deposition, whereas inflammatory CAF programs may respond better to modulation of IL-6, JAK/STAT, CXCL12, or TGF-β signaling. Sparing physiological fibroblasts is possible through dual-marker recognition, protease- or pH-gated release, intratumoral administration, and transient reprogramming instead of cell killing. Such safeguards need to be tested in wound-healing and non-tumor fibrotic models, not tumor-bearing mice alone (8, 11, 19, 20). Oxygenation and treatment sequencing data should likewise be read against this CAF-state biology rather than as generic stromal-depletion results (8, 10, 11, 15). Recent CAF therapy literature reinforces the same context-specific view (12).

### 2.1 Fibroblast-targeted nanoparticles

The shift to fibroblast-selective nanoparticles changes the goal from directly targeting tumor cells to controlling the surrounding stroma. FAP-binding peptides, antibodies, small molecules, and protease-activated constructs can concentrate payloads in CAF-rich regions, whereas collagen- and tenascin-directed carriers engage the surrounding matrix. The strength of this approach is also its main hazard: FAP and related stromal programs appear in wound healing and in some normal tissues, and depletion studies show that removing stroma is not always helpful. Therefore, designs should lean toward local activation, reversible reprogramming of the CAF state, or disease-enriched marker combinations rather than blanket CAF ablation (23, 24). This caution echoes the wider CAF-therapy literature, which stresses that benefits and harms are context-dependent (12).

Collagen-binding and matrix-responsive carriers can boost retention in collagen-rich regions; however, a strong affinity for the matrix can also form a binding-site barrier that blocks deeper diffusion (25, 26). MMP-cleavable linkers offer another route, translating protease activity into changes in size, charge, and payload release. MMP responsiveness is not a generic tumor signal; soluble MMP-2 and MMP-9 and membrane-bound MMP-14 differ in cellular source, localization, and substrate preference. Therefore, linker sequences need to be profiled across the relevant isoforms and in non-tumor inflammatory or remodeling tissues to limit premature cleavage off target (27). Recent reviews of MMP-responsive delivery systems have established useful design criteria for protease-gated carriers (27).

Interpreting nanoparticle penetration and clearance requires a consistent set of parameters—hydrodynamic diameter, PDI, zeta potential, morphology, ligand and PEG density, drug loading or encapsulation efficiency, and development stage; however, reporting was uneven across the cited studies. Where a value was not reported, we have not inferred it. As **Table 1** makes clear, nanoparticle design keeps running into the same tension: the features that favor long circulation and tumor retention often work against deep tissue penetration. Particles in the tens-to-low-hundreds of nanometers are large enough to escape rapid renal clearance, yet smaller or size-transformable systems usually diffuse more readily through the dense, poorly permeable stroma of collagen-rich tumors. Shape and mechanics matter just as much: rod-shaped or deformable particles marginate toward vessel walls and are internalized differently from spheres; strongly cationic surfaces boost cellular association but also draw protein adsorption and faster mononuclear-phagocyte clearance; and thick PEG layers prolong circulation yet can bury targeting ligands and dampen uptake. Taken together, size, shape, charge, PEG architecture, ligand density, and loading efficiency behave as coupled variables rather than knobs that can be tuned in isolation (25, 28–30). Pharmacokinetic and intratumoral-penetration studies reinforce this: these parameters act as linked determinants of delivery, not as isolated formulation descriptors (26).

**Table 1:** CAF- and matrix-directed nanomedicine strategies and reporting priorities.

Target	Ligand/platform strategy	Physicochemical reporting and design variables	Development stage/application
FAPα (23, 24, 31)	FAP-binding ligands on liposomal or polymeric carriers	Hydrodynamic diameter, PDI, zeta potential, ligand and PEG density, and encapsulation efficiency should be reported; values are formulation-specific and were not consistently available across the cited studies.	Predominantly preclinical; CAF modulation, stromal normalization, or payload localization
αSMA/myCAF programs (8, 11, 19, 20)	Myofibroblast-directed or TGF-β-pathway reprogramming	Intracellular αSMA is not an ideal surface ligand; carrier size, tissue penetration, and local release selectivity are critical to avoid non-tumor myofibroblasts.	Preclinical; CAF-state reprogramming rather than indiscriminate depletion
Collagen-rich ECM (4, 25, 26)	Collagen-binding or matrix-responsive carriers	Smaller or size-transformable particles generally penetrate dense ECM more effectively, whereas excessive matrix affinity can produce a binding-site barrier.	Preclinical; stromal retention and improved intratumoral distribution
PDGFRα/stromal receptors (8, 11, 22)	Receptor-directed silica, lipid, or polymeric systems	The surface ligand density and charge must be balanced against protein adsorption, MPS clearance, and off-tumor stromal uptake.	Preclinical; stromal targeting and co-delivery
Tenascin-C/stromal ECM (21, 22)	Tenascin-C-binding peptides or aptamers	Multivalent avidity can improve stromal retention; however, particle size and ligand density should be optimized to preserve diffusion.	Preclinical; stromal homing and localized drug delivery
MMP-responsive release (27)	Protease-cleavable linkers in micelles, hydrogels, or polymeric nanoparticles	The linker sequence should be validated against MMP-2, MMP-9, and membrane-associated MMP-14, including cleavage in normal inflammatory or remodeling tissues.	Preclinical; stimulus-responsive payload release

## 3 Hypoxia-responsive nanomedicine systems

**Figure 2:**
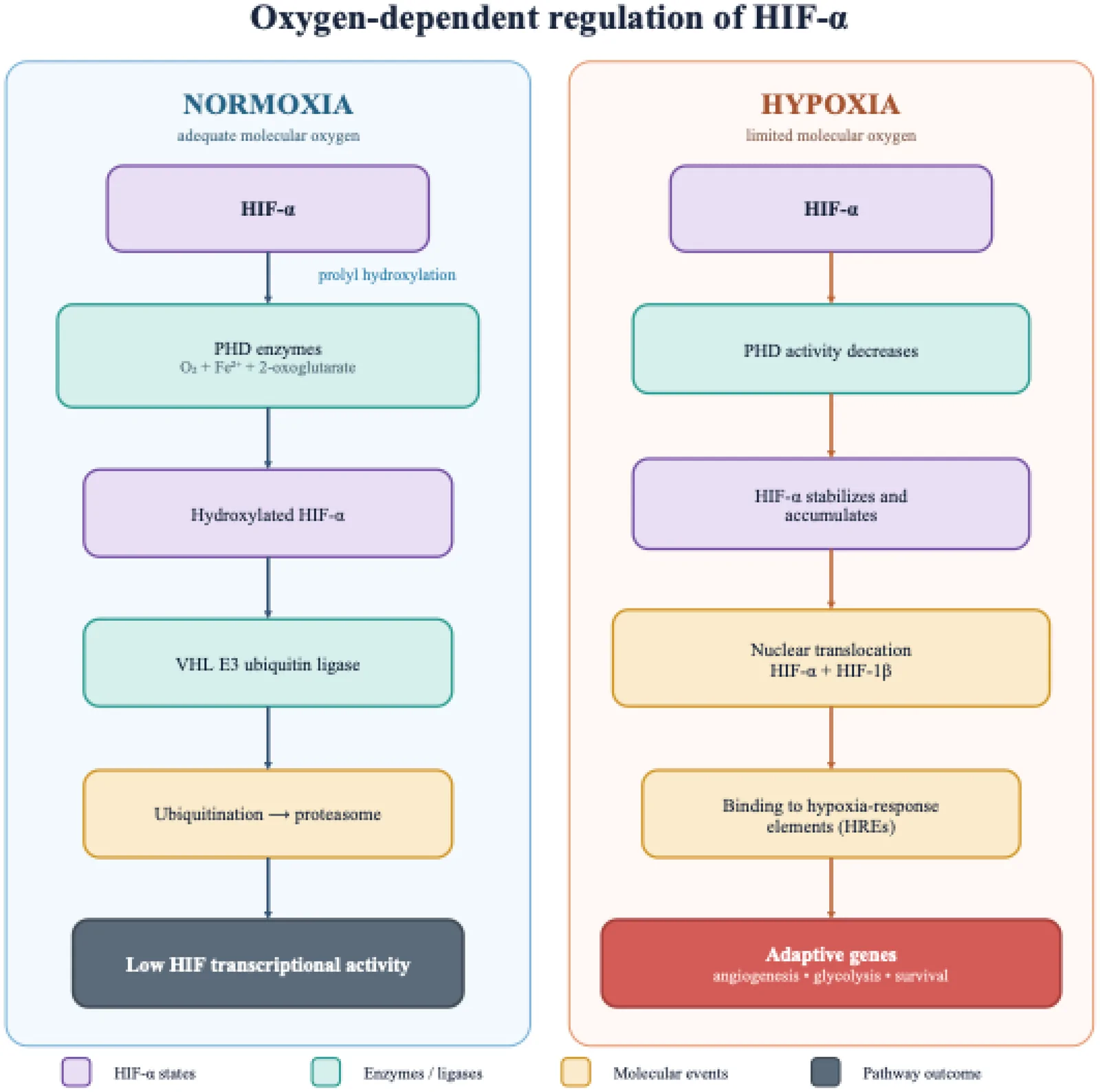
Canonical regulation of hypoxia-inducible factors under normoxic and hypoxic conditions. Under normoxia, prolyl hydroxylase domain enzymes hydroxylate HIF-α, enabling von Hippel-Lindau-mediated ubiquitination and proteasomal degradation. Under hypoxia, reduced hydroxylation permits HIF-α stabilization, nuclear translocation, dimerization with HIF-1β, and transcriptional activation of the adaptive programs. Conceptual schematic based on the canonical PHD-VHL-HIF pathway (32).

PDT is a useful proving ground for TME-targeted nanomedicine because its efficacy depends on the three things nanoparticles are built to address: stromal density, oxygen supply, and immune suppression. Hypoxia is at the center of this problem. It is a defining feature of the tumor microenvironment and a major obstacle to treatment efficacy. In physiological terms, hypoxia means tissue oxygen tension well below normal, typically under 5–10 mmHg in tumors versus 40–60 mmHg in healthy tissue. This imbalance is caused by abnormal, poorly perfused vasculature, which restricts oxygen delivery, while rapidly dividing cancer cells consume more oxygen. The hypoxic niche weakens radiotherapy, PDT, and several chemotherapies. HIF signaling, chiefly HIF-1α and HIF-2α, is at the core of the cellular response. HIF-1α is often considered the master regulator of low-oxygen gene expression; however, the isoforms are not interchangeable. Under normoxia, prolyl hydroxylase domain (PHD) enzymes continuously hydroxylate HIF-1α, marking it for von Hippel-Lindau (VHL)-mediated ubiquitination and proteasomal degradation. When oxygen levels fall, PHD activity drops, and HIF-1α stabilizes, accumulates, and dimerizes with HIF-1β. The complex enters the nucleus, binds hypoxia-responsive elements (HREs) in target gene promoters, and drives more than 100 downstream effectors associated with angiogenesis (VEGF), metabolic reprogramming (GLUT1 and LDHA), invasion (MMPs), and treatment resistance. **Figure 2** summarizes the canonical oxygen-dependent regulation of HIF-α through the PHD-VHL pathway (5, 32). Hypoxia is particularly detrimental to PDT. Conventional PDT depends on molecular oxygen to produce cytotoxic reactive oxygen species (ROS) once light activates the photosensitizer, mainly through Type II energy transfer from the triplet-state photosensitizer to ground-state oxygen, yielding singlet oxygen. In areas where oxygen is scarce, as is usually the case in tumors, this pathway falters, and its efficacy decreases. Worse, PDT can burn through local oxygen faster than it is resupplied, deepening hypoxia in already compromised tissues (16). Nanomedicine offers several complementary solutions. One approach involves generating or delivering oxygen directly, thereby increasing local availability and restoring the sensitivity of hypoxic tumors to oxygen-dependent therapy. The second step involves turning the hypoxic environment into a trigger for site-specific release, reading out biochemical markers such as elevated nitroreductase activity, low pH, and high HIF-1α. A third lesson —without abolishing—oxygen dependence by favoring oxygen-sparing or low-oxygen-tolerant Type I photochemistry, which produces radical species through electron- or hydrogen-transfer (10, 33–35). Hypoxia-responsive carriers can be built in reductase-sensitive azo or nitroaromatic groups that change their chemical state at low oxygen levels and, in doing so, alter carrier solubility or release the payload. Because both reductase activity and oxygen tension vary between tumors and normal tissues, activation thresholds, off-target cleavage, and release kinetics should be measured directly rather than assumed from the linker chemistry on paper (33).

HIF-2α deserves attention, next to HIF-1α. The two isoforms share HIF-1β and the same oxygen-dependent regulation; however, their transcriptional programs are non-redundant and sometimes opposing, differing by tumor and cell type. HIF-1α governs acute hypoxic adaptation and glycolysis, whereas HIF-2α drives long-term adaptation, angiogenesis, stemness, and immunosuppressive myeloid states. The selective HIF-2α inhibitor belzutifan has shown that isoform-specific targeting works in susceptible diseases; however, this does not mean that HIF-2α blockade will help across all solid tumors. Nanocarriers might increase local exposure to isoform-selective or dual-HIF cargoes, although biomarker selection and tumor-context testing remain essential (15).

Type I and Type II PDT respond to hypoxia in complementary ways. Type II photosensitization transfers energy to molecular oxygen to produce singlet oxygen; therefore, it is strongly oxygen-dependent. Type I systems use electron or hydrogen transfer to generate radicals, such as superoxide and hydroxyl; they are not oxygen-free but are active at lower oxygen tensions. Nanoparticles can bias a design toward the Type I route by controlling photosensitizer aggregation, donor-acceptor engineering, redox-active components, or hypoxia-activated switching. Any comparison should report oxygen tension, illumination dose, ROS identity, dark toxicity, and depth of response, since efficacy measured under different conditions cannot be compared directly (16, 17, 34). Recent activatable Type I photosensitizers have made it possible to explicitly state the ROS identity and oxygen tension (34).

### 3.1 Stromal hypoxia modulation: linking CAF activity to oxygen status

The link between stromal fibroblasts and tumor oxygenation is a key aspect of microenvironment-aware design. Contractile myCAFs, working through a collagen-rich matrix, compress tumor vessels and cut blood flow and oxygen delivery, most visibly in desmoplastic tumors such as pancreatic cancer. CAF-secreted VEGF and other pro-angiogenic factors add leaky, disorganized vessels that further impair oxygenation. The relationship runs both ways: hypoxia stabilizes HIF signaling in tumor cells and CAFs, reinforcing contractility, matrix deposition, glycolysis, lactate production, and vascular dysfunction. Stromal normalization and oxygen-generating carriers have improved perfusion or oxygenation in individual preclinical models; however, the reported magnitudes cannot be pooled because the studies differ in tumor model, oxygen probe, imaging window, and denominator. Comparisons should instead report baseline and post-treatment pO_2_ or hypoxic fraction using one validated method, not a single cross-study percentage (4, 8, 10, 15).

Preclinical oxygen-modulating nanoparticles follow several distinct routes and should not be flattened into one claim of CAF-selective oxygen delivery. Albumin-MnO_2_ carriers exploit acidic, H_2_O_2_-rich conditions to dissociate and release oxygen, and perfluorocarbon@porphyrin nanoparticles physically ferry oxygen to support PDT (10, 35). Neither marker alone demonstrated FAP-dependent stromal localization. A genuine CAF/hypoxia co-targeting platform must demonstrate receptor-dependent accumulation in CAF-rich regions, quantify oxygenation using a validated method, and include both non-targeted and non-oxygenating controls. Acid-labile linkers can tie release to local pH; however, acidity is not unique to tumors and must be checked in normal inflammatory and wound-healing tissues.

Stromal access and oxygen supply are coupled constraints in PDT; therefore, **Table 2** separates oxygen generation, oxygen delivery, hypoxia-triggered release, HIF pathway modulation, and CAF/hypoxia co-targeting concepts. The last row represents a design concept, not a proven cross-study platform; testing it will require standardized oxygen measurements and direct evidence of CAF-selective localization.

**Table 2:** Hypoxia-modulating nanomedicine strategies and translational priorities.

Strategy	Mechanism	Nanoparticle platform	Key advantage and translational priority
O_2_ generation (10)	MnO_2_-catalyzed conversion of endogenous H_2_O_2_	pH-/H_2_O_2_-responsive albumin-MnO_2_ nanoparticles	On-demand O_2_ production; efficacy depends on endogenous H_2_O_2_, acidity, catalytic persistence, and tumor distribution
O_2_ delivery (35)	Physical transport of dissolved O_2_	Perfluorocarbon@porphyrin nanoparticles	High O_2_ capacity; loading, leakage, vascular access, and release kinetics remain limiting
Hypoxia-triggered release (33)	Low-oxygen-dependent chemical conversion	Hypoxia-responsive albumin-based nanosystem	Site-specific activation; requires a validated hypoxia biomarker and sufficient trigger activity
pH-/H_2_O_2_-responsive dissociation (10)	Acidic and oxidative TME cues change carrier structure	Albumin-MnO_2_ nanocarrier	Couples carrier disassembly with oxygen modulation, but acidic pH and H_2_O_2_ are not tumor-exclusive
HIF-pathway modulation (15)	Isoform-selective or context-specific pathway inhibition	siRNA or small-molecule nanocarrier (design class)	Direct pathway modulation; HIF-1α and HIF-2α have non-redundant, tumor-dependent functions
CAF/hypoxia co-targeting (8, 10, 15)	CAF-state modulation combined with a validated oxygen-modulating module	Modular CAF-selective carrier plus oxygen-generating cargo (design concept)	Potentially links stromal decompression and oxygen restoration; requires direct proof of CAF selectivity and standardized pO_2_ measurement

Hypoxia-responsive systems must balance oxygen generation, trigger sensitivity, and controlled release. Albumin-MnO_2_ nanoparticles and hypoxia-responsive albumin carriers exhibit two distinct elements: oxygen modulation and low-oxygen triggering (10, 33). Combining both in a single carrier is still a testable hypothesis and not an established gain. Comparative studies should include independent non-oxygenating and non-responsive controls and quantify oxygenation, activation, and release using validated assays.

## 4 Immune modulation in the tumor microenvironment

The real challenge in cancer immunotherapy is not switching immunity on, but keeping it on inside a tumor microenvironment that is metabolically hostile, structurally obstructive, and actively suppressive. Checkpoint blockade, co-stimulatory agonism, innate immune activation, cytokine engineering, and local delivery each address different aspects of this problem. Nanoparticle-enabled PDT is distinguished by the combination of local tumor destruction and immunogenic cell death with the spatially controlled release of immunomodulators, transforming a localized event into systemic antitumor immunity (9, 13, 14). Effective immune modulation, then, is about reversing the suppressive dynamics of the microenvironment, not simply amplifying immunity. In many solid tumors, chronic antigen exposure pushes CD8 T cells toward exhaustion, with more inhibitory receptors and less cytokine output, while regulatory T cells (Tregs), myeloid-derived suppressor cells, and M2-like/tumor-associated macrophages entrench tolerance through IL-10, TGF-β, arginase activity, and suppressive cell-cell contact. Hypoxia, lactate, adenosine, glucose depletion, and disordered vasculature pile on, throttling effector lymphocytes, and blocking sustained immune cell trafficking (36–39). Mechanistic details are important for design: PD-1 blockade works best when a stem-like, TCF1-positive exhausted CD8 pool is still present, since it expands and differentiates that population—but on its own, it cannot overcome stromal exclusion, myeloid suppression, or vascular dysfunction (40–42). These principles apply to both the promise and limitations of checkpoint inhibitors. Blocking PD-1/PD-L1 and CTLA-4 has reshaped clinical oncology; however, the benefit concentrates in tumors that already carry at least a partial immune infiltrate. In advanced melanoma, nivolumab plus ipilimumab achieved a 5-year overall survival of 52%, compared to 44% for nivolumab alone, which is a benchmark for deep, durable responses, albeit with added toxicity (43). In metastatic non-small cell lung cancer with high PD-L1 expression, first-line pembrolizumab raised the 6-month overall survival to 80.2% from 72.4% with platinum chemotherapy, improved response rates, and reduced severe treatment-related adverse events (44). The next wave of checkpoint work targets additional exhaustion-associated receptors. In untreated melanoma, relatlimab plus nivolumab stretched the median progression-free survival to 10.1 months from 4.6 months with nivolumab alone, a reminder that resistance often rests on a network of inhibitory signals rather than PD-1 alone (45). Immune-related toxicity, primary resistance in cold tumors, and partial responses remain real limitations. Co-stimulatory agonists aim to supply the positive signals that checkpoint blockade cannot, such as the priming, expansion, and survival cues required for antitumor T cells. CD137/4-1BB and OX40 boost T-cell proliferation, effector function, and memory, while CD40 activates dendritic cells and macrophages to enhance antigen presentation and T-cell priming. Clinically, this class has been difficult to translate because systemic antibodies often have a narrow therapeutic window or weak single-agent activity. In the ENGAGE-1 phase I study, the OX40 agonist GSK3174998, with or without pembrolizumab, engaged its target but did not show sufficient clinical activity to justify further development (46). CD40 agonism has been more effective in immunologically cold diseases. In the randomized phase II PRINCE trial in metastatic pancreatic cancer, nivolumab plus chemotherapy met the primary 1-year overall survival endpoint at 57.7%, while sotigalimab plus chemotherapy reached 48.1% against a 35% historical benchmark, with each arm showing its own immune correlates (47). The lesson is that co-stimulation works best alongside antigen release, myeloid reprogramming, or improved local delivery rather than alone (47, 48). Innate agonists and cytokines face a different bottleneck: insufficient priming. Toll-like receptor and STING agonists can mature dendritic cells, launch type I interferon programs, and turn minimally inflamed lesions into sites of T-cell priming—but delivery makes or breaks them. Intratumoral TLR9 agonism with SD-101 plus pembrolizumab produced high response rates in a small, anti-PD-1-naive melanoma cohort, but fell off sharply in patients previously treated with anti-PD-1, with injection-site and flu-like reactions dominating the safety profile (49). The STING agonist MIW815/ADU-S100 with spartalizumab was similarly manageable but delivered few objective responses across advanced cancers, underscoring that pharmacology, lesion access, and intratumoral distribution weigh as heavily as pathway choice (50). Cytokines encounter the same wall. Although systemic cytokine engineering is appealing in theory, a phase III trial of bempegaldesleukin plus nivolumab failed to improve outcomes in untreated melanoma (51). The field is moving toward local cytokine production: intratumoral lipid nanoparticles carrying IL-21, IL-7, and 4-1BBL mRNA sharply increased tumor-infiltrating, granzyme B-positive, IFN-γ-producing CD8 T cells and built memory responses across several mouse models, beating anti-PD-1 alone in some settings (52).

**Figure 3:**
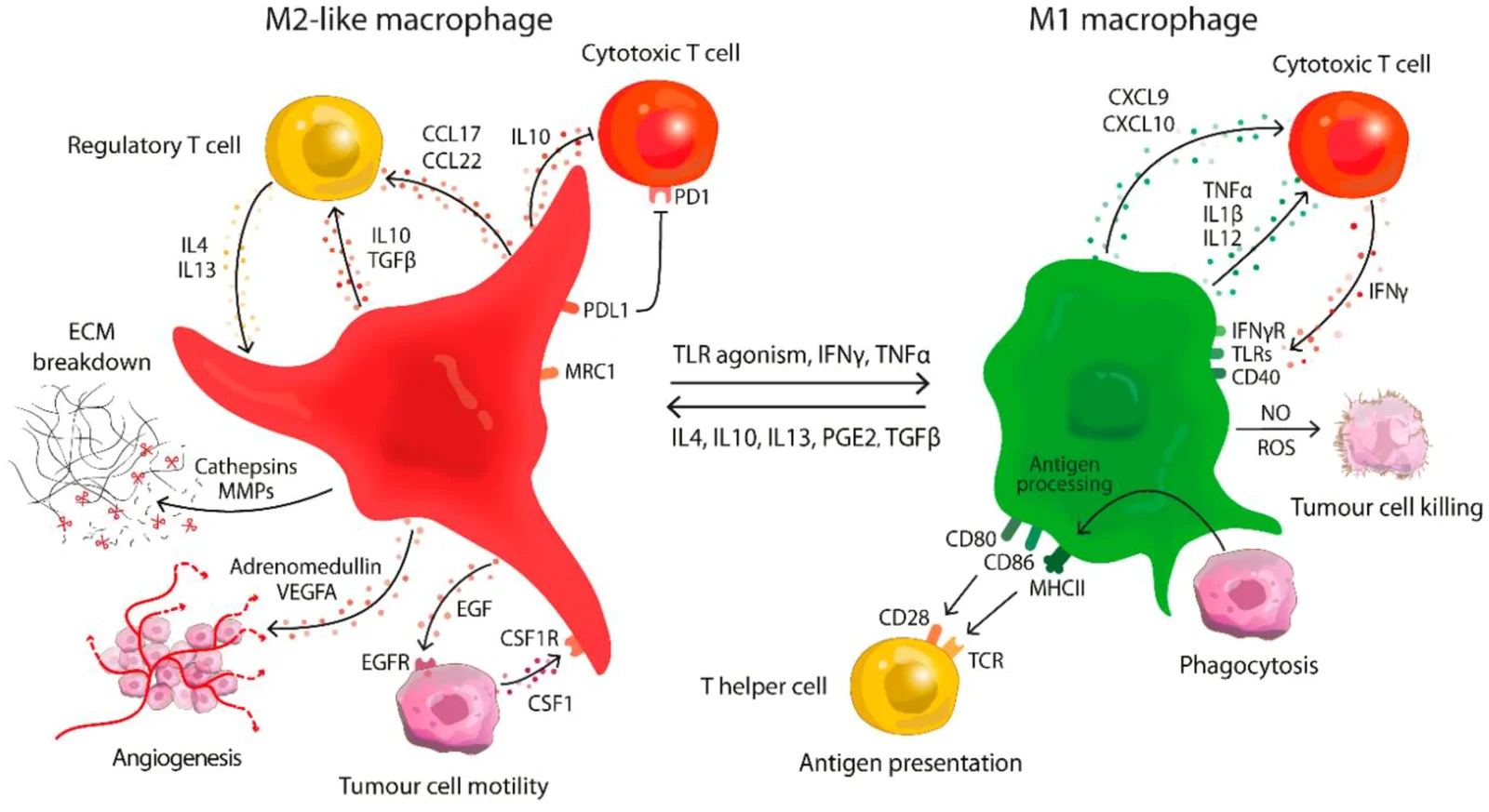
Macrophage polarization states in the tumor microenvironment. M2-like tumor-associated macrophages (TAMs, red) promote immunosuppression through IL-10, TGF-β, and PD-L1 signaling, thereby suppressing cytotoxic T-cell activity. M1-like macrophages (green) support antigen presentation and proinflammatory effector functions. Nanoparticle-based reprogramming strategies aim to shift TAM functional states while recognizing that the M1/M2 framework is a simplification of a broader continuum. Conceptual schematic based on the macrophage-targeting literature (39).

The pattern that emerges is combinatorial and spatially aware: lift inhibitory signaling, add co-stimulation, trip innate sensing, and deliver those cues where the antigen is actually being released. This is why delivery has moved to the center of immune modulation rather than to the margins. Vascular and immune normalization can improve trafficking and ease hypoxia; local RNA and nanoparticle systems can concentrate agonists in the tumor while sparing the rest of the body; and immunogenic therapies can generate the antigenic substrate on which checkpoint or co-stimulatory agents need to act. The future lies in sequential or co-delivered regimens that convert non-inflamed tumors into lesions capable of dendritic cell activation, T-cell priming, intratumoral expansion, and lasting memory. This is the bridge to nanoparticle-enabled photodynamic therapy, where local oxidative killing is deliberately coupled with programmed immune amplification (9, 13, 14, 52). The macrophage polarization states and their functional consequences in the immunosuppressive microenvironment are shown in **Figure 3**.

The suppressive myeloid compartment does not stop at the TAMs. Monocytic and polymorphonuclear MDSCs restrain T cells through arginase-1, nitric oxide, reactive oxygen species pathways, nutrient depletion, and suppressive cytokines (7, 37). Nanomedicine might counter them by blocking recruitment of suppressive cells or by delivering reprogramming cargoes into myeloid-rich niches; macrophage-directed mRNA and siRNA nanocarriers are representative preclinical examples (53, 54). Tumor-associated neutrophils are equally plastic. The N1/N2 labels are a convenient shorthand for anti-versus pro-tumor phenotypes; however, TANs and polymorphonuclear MDSCs overlap and cannot be cleanly separated by that binary. Any strategy that redirects neutrophil trafficking or uses neutrophils as carrier cells must distinguish transient delivery from durable immunosuppressive reprogramming and monitor infection risk. TAN targeting has its own literature and should not be folded into a generic TAM framework (55).

### 4.1 Nanoparticle-enabled photodynamic immunotherapy

Photodynamic therapy is well-suited for immunotherapy because it does more than just debulk tumors. Upon light activation, photosensitizers generate reactive oxygen species that can induce immunogenic cell death, releasing calreticulin, ATP, HMGB1, and tumor antigens that recruit and prime dendritic cells. Conventional photodynamic therapy (PDT) presents challenges, including the potential exacerbation of hypoxia, induction of compensatory signaling pathways such as PD-L1, STAT3, or IDO, and inadequate engagement with distant disease sites. Nanoparticles address this challenge by co-localizing photosensitizers with immunomodulators, ensuring concurrent antigen release with checkpoint inhibition or adjuvant signaling. This approach also enhances tumor retention and penetration (56, 57).

In these studies, a consistent finding emerged: the antigen generated by PDT must be accompanied by a secondary immune instruction. **Table 3** is arranged by combination class (chemo-PDT, checkpoint or metabolic blockade, innate adjuvants, and nucleic-acid systems), with each row citing its source. Abscopal responses remain inconsistent because the models differ in tumor antigenicity, contralateral tumor timing, illumination geometry, photosensitizer dose, immune cargo sequence, and even how a distant response is defined. Until reports include both irradiated and non-irradiated lesions, systemic immune correlates, and rechallenge outcomes, cross-platform claims cannot be compared.

**Table 3:** Comparative summary of representative nanoparticle–PDT–immunotherapy studies.

Combination class/reference	Nanoparticle	Payloads	Tumor model	Immune outcomes
Chemo-PDT + checkpoint blockade (56)	Core-shell nanoscale coordination polymer	Oxaliplatin core + pyrolipid shell; combined with anti-PD-L1	Bilateral CT26/MC38 colorectal cancer	CRT exposure, tumor-specific IFN-γ T cells, increased CD8 infiltration, abscopal regression
PDT + metabolic checkpoint inhibition (65)	Liposomal conjugate	Porphyrin photosensitizer + IDO inhibitor NLG919	Bilateral 4T1 breast cancer	ICD plus IDO blockade, stronger CD8 responses in primary and distant tumors
Chemo-PDT + checkpoint blockade (66)	Light-triggered prodrug nanoparticle	Chemotherapy prodrug + PDT module; combined with PD-L1 blockade	Syngeneic murine tumor models	Converted low-immunogenic tumors toward immune activation, reduced recurrence and lung metastasis
PDT + local checkpoint silencing (67)	Nucleic-acid nanogel	Photosensitizer + PD-L1 siRNA	Bilateral mouse tumor model	Local checkpoint silencing with enhanced photoimmunotherapy and distant-tumor control
PDT + aptamer/adjuvant signaling (68)	Photocontrolled DNA nanomedicine	Photosensitizer + PD-L1 aptamer + CpG	Murine tumor model	Light-gated adjuvant/checkpoint release, enhanced membrane-targeted PDT immunotherapy
Biomimetic oxygenation + checkpoint blockade (69)	Biomimetic hybrid dual-targeting nanoparticle	Dual-enzyme nanozyme + hybrid membrane coat + tumor-targeting antibody; combined with anti-PD-1	Deep glioblastoma, including postsurgical recurrence	Oxygenation, ECM remodeling, macrophage repolarization, synergy with anti-PD-1
Type I PDT + STAT3/HIF-1α modulation (57)	Hypoxia-tolerant polymeric photosensitizer prodrug	Type-I polymeric photosensitizer + niclosamide (STAT3 inhibitor)	4T1 breast cancer with distant-tumor and rechallenge settings	Higher DC maturation, higher CD8/Treg ratios, reduced M2/HIF-1α signaling, improved survival

Across representative studies, three design principles have recurred: co-localization of PDT with immune modulation, use of tumor-responsive release to widen the therapeutic index, and engineering systemic immunity from a local, light-triggered event (56, 65, 67, 69).

**Figure 4:**
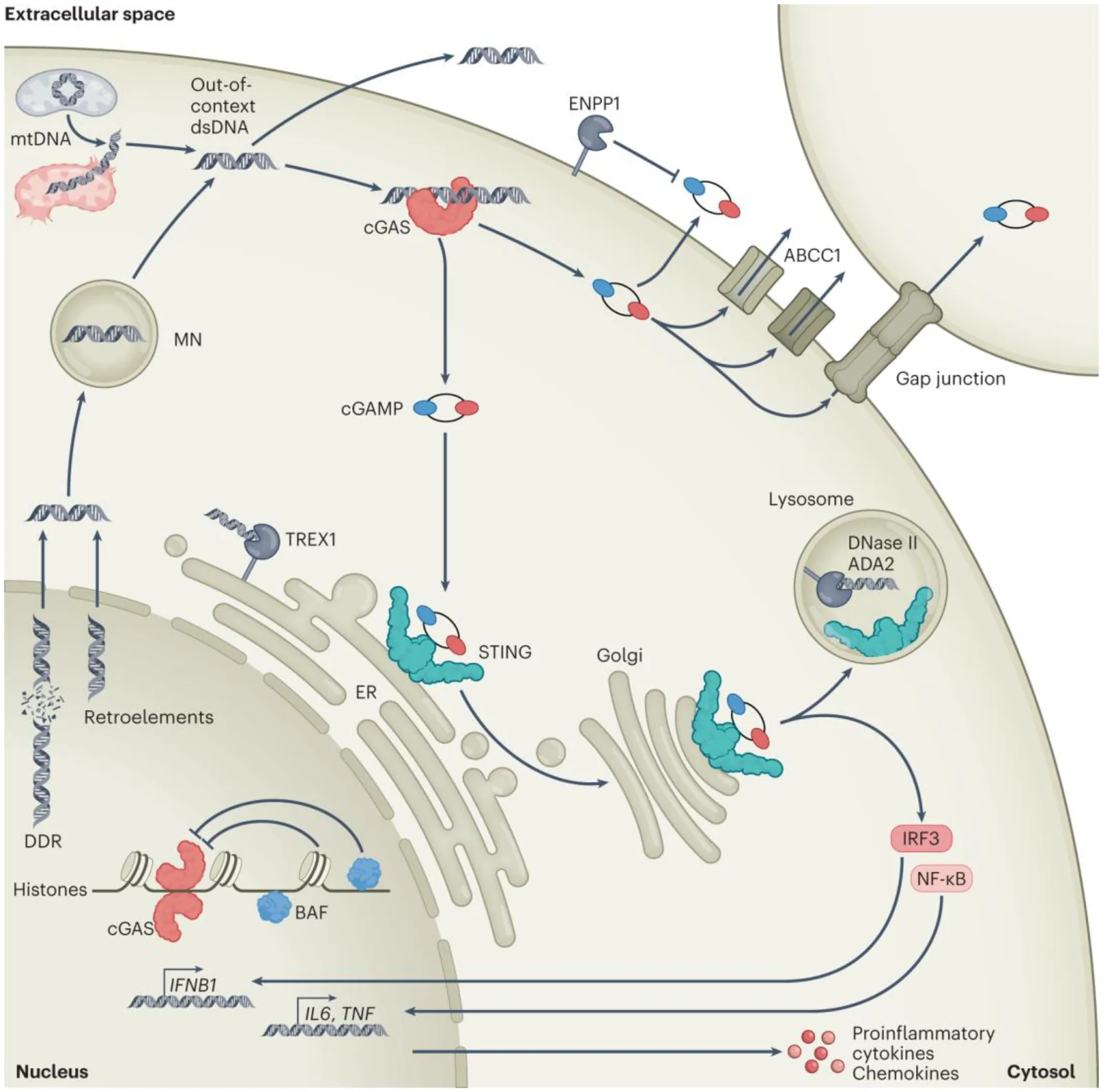
cGAS-STING cytosolic DNA-sensing pathway. Cytosolic DNA from tumor cells, mitochondria, or viral sources is detected by cGAS, which synthesizes cyclic GMP-AMP (cGAMP). cGAMP binds to STING on the ER membrane, triggering downstream signaling through TBK1 and IRF3 to induce type I interferon production. Nanoparticle platforms enable the targeted delivery of STING agonists to tumor sites, amplifying innate immune activation. Adapted from Samson and Ablasser (58).

The cGAS-STING cytosolic DNA-sensing pathway and its nanoparticle-enabled activation for cancer immunotherapy are shown in **Figure 4**.

The translational read is encouraging but is guarded. In mice, integrated nanoparticle-PDT regimens reliably improve local control, dendritic cell activation, CD8 expansion, and abscopal immunity; however, almost all of this work is still preclinical. Getting to the clinic will require simplifying architectures, matching light physics to the disease site, and designing combination trials around biomarkers of ICD, myeloid reprogramming, and T-cell priming rather than tumor shrinkage alone. Deep or irregularly shaped lesions require a defined optical delivery plan. Endoscopic, intraoperative, or interstitial fiber-optic illumination can bring light close to the target area. NIR and emerging NIR-II photosensitizers extend the usable depth but do not erase scattering and dosimetry limits. Implantable or externally powered micro-light sources remain experimental (16, 59). Radiation- and ultrasound-triggered systems are separate modalities and should not be labeled as conventional PDT. Implantable micro-light-source work shows an experimental route for deep or spatially constrained lesions (59). Representative nanoparticle-PDT-immunotherapy studies are compared in **Table 3**.

The mRNA vaccine experience shows how delivery constraints are tracked with disease. In resected melanoma, V940 primes systemic neoantigen-specific immunity in a setting already sensitive to checkpoint blockade; therefore, direct nanoparticle penetration into a bulky desmoplastic mass is not the whole story. In PDAC, autogene cevumeran acts as a vaccine; however, the residual or recurrent niche is denser in stroma, richer in myeloid cells, and colder immunologically. Published clinical reports do not provide sufficient formulation details for a rigorous head-to-head comparison of LNP compositions. The comparison that holds up is biological: antigen quality, route, access to lymphoid organs and APCs, stromal exclusion, and the surrounding checkpoint or chemotherapy context all differ between melanoma and PDAC (60–64).

### 4.2 Clinical translation: from preclinical promise to therapeutic reality

After two decades of intensive work and strong preclinical signals, only a handful of cancer nanomedicines have been approved, and most of them improve the pharmacokinetics or tolerability of existing drugs rather than reprogramming the TME. Broad pan-cancer reviews offer helpful catalogs of platforms and trials (18), but the translational gap makes more sense mechanistically: variable vascular access, stromal exclusion, protein-corona formation, off-target clearance, manufacturing complexity, and weak biomarker selection can each wipe out an apparent preclinical advantage.

The enhanced permeability and retention (EPR) effect is not a uniform delivery mechanism in the body. A quantitative analysis of preclinical studies reported that the median tumor delivery was approximately 0.7% of the injected nanoparticle dose, with a wide variation across models and platforms. In PDAC, dense desmoplasia, high interstitial pressure, and compressed vasculature narrow extravasation and diffusion. The takeaway is to measure the delivery and TME state in each trial rather than assume passive accumulation (26, 70, 71).

A central obstacle is the protein corona that forms when nanoparticles meet blood. Adsorbed proteins can mask targeting ligands, change cellular uptake, activate complement, and steer particles toward the liver and spleen. PEGylation reduces nonspecific adsorption and lengthens circulation; however, its success depends on chain length and surface density, and it can blunt receptor engagement or provoke anti-PEG responses. Zwitterionic surfaces, biomimetic membrane coatings, alternative hydrophilic polymers, and deliberately pre-formed coronas all appear promising, yet none are universally non-fouling. Therefore, preclinical testing should measure particle size and zeta potential before and after incubation in relevant human plasma or serum, add corona proteomics where feasible, and check complement activation and donor-to-donor variability under flow (72). Manufacturing has its own demands: GMP control must span size distribution, PDI, morphology, surface composition, free versus encapsulated drug, potency, sterility, stability, and batch comparability, with extra controls for every targeting ligand or responsive element.

The clinical track record of approved nanomedicines is instructive for future TME-targeted platforms. Doxil and Abraxane demonstrate that nanomedicine can be clinically valuable by improving pharmacokinetics, safety, or tolerability, even without reprogramming the TME. Patient stratification is central to selecting the cancers most likely to respond to microenvironment-targeted therapy. Tumors with high FAP expression, collagen accumulation, abundant hyaluronan, or validated hypoxia signatures may be the best candidates for stromal or oxygen-responsive strategies. Future trials should build in prespecified biomarker thresholds and pharmacodynamic TME endpoints so that a measured change in the microenvironment can be tied to efficacy rather than inferred from tumor shrinkage.

Failed phase III trials set some of the most stringent design constraints. In HA-high metastatic PDAC, HALO-301 found that adding PEGPH20 to nab-paclitaxel/gemcitabine raised the response rate but did not improve overall survival (11.2 versus 11.5 months; HR 1.00) or progression-free survival (7.1 months in both arms), proving that a plausible stromal biomarker and a matrix-depleting mechanism do not guarantee benefit (73). In MAESTRO, the hypoxia-activated prodrug evofosfamide with gemcitabine missed its primary overall survival endpoint (8.7 versus 7.6 months; HR 0.84; P = .059) despite encouraging phase II activity (74). Taken as a pair, they argue for validated spatial biomarkers, direct confirmation of target engagement or prodrug activation, respect for the context-dependent protective roles of stroma, and early testing of whether a mechanism actually improves delivery at metastatic and primary sites.

The implementation of TME-targeted nanomedicine requires a balance between scientific ambition and clinical feasibility. Near-term progress will most likely come from focused platforms that solve a specific microenvironmental problem, such as stromal normalization or hypoxia relief, inside a well-defined patient group, rather than from elaborate systems that attempt to modulate every major component of the TME simultaneously. The longer game depends on reliable biomarkers for patient selection, scalable manufacturing, and trial designs that read out microenvironmental endpoints, alongside conventional efficacy. Together, these steps provide nanomedicine with the best chance of delivering its therapeutic promise in cancer. The selected clinical studies relevant to immune modulation, stromal targeting, and hypoxia targeting are listed in **Table 4**; the registry statuses were verified on July 10, 2026.

**Table 4:** Selected clinical studies relevant to immune modulation, stromal targeting, and hypoxia-targeting.

Trial (NCT)	Phase	Intervention/platform	Indication	Registry status	Published finding or current limitation
KEYNOTE-942 (NCT03897881) (60, 61)	2	Intismeran autogene (V940/mRNA-4157) + pembrolizumab	Resected high-risk melanoma	Active, not recruiting (updated Dec 2025)	A randomized phase 2b report showed improved recurrence-free survival, and long-term registry follow-up is ongoing.
V940-001/INTerpath-001 (NCT05933577)	3	Intismeran autogene (V940) + pembrolizumab vs placebo + pembrolizumab	Resected high-risk melanoma	Active, not recruiting (updated Sep 2025)	No phase 3 efficacy results have been posted, and primary completion is projected for 2029.
V940-002/INTerpath-002 (NCT06077760)	3	Intismeran autogene (V940) + pembrolizumab vs placebo + pembrolizumab	Resected NSCLC	Recruiting (updated Jul 2026)	No efficacy results have been posted, and primary completion is projected for 2030.
IMCODE003 (NCT05968326) (62, 63)	2	Autogene cevumeran + atezolizumab + mFOLFIRINOX vs mFOLFIRINOX	Resected PDAC	Recruiting (updated Jul 2026)	The cited phase 1 study established vaccine-induced T-cell responses, and the randomized phase 2 efficacy study is ongoing.
RELATIVITY-047 (NCT03470922) (45)	2/3	Nivolumab + relatlimab vs nivolumab	Untreated unresectable or metastatic melanoma	Active, not recruiting (updated Sep 2025)	Published primary analysis: median PFS 10.1 vs. 4.6 months; registry follow-up continues.
ECHO-301/KEYNOTE-252 (NCT02752074) (75)	3	Epacadostat + pembrolizumab vs placebo + pembrolizumab	Unresectable or metastatic melanoma	Completed (updated Aug 2025)	It failed to improve progression-free or overall survival, cautioning against single-node metabolic targeting.
MIW815/ADU-S100 (NCT02675439) (50, 76)	1	Intratumoral STING agonist, alone or with checkpoint blockade	Advanced solid tumors or lymphomas	Terminated (updated Dec 2021)	Published studies have shown target engagement but limited objective activity, emphasizing delivery and lesion-access constraints.
HALO-301 (NCT02715804) (73)	3	PEGPH20 + nab-paclitaxel/gemcitabine vs placebo combination	Hyaluronan-high metastatic PDAC	Terminated (updated Jul 2020)	No OS or PFS benefit was observed despite biomarker enrichment and a plausible matrix-depletion mechanism.
MAESTRO (NCT01746979) (74)	3	Evofosfamide + gemcitabine vs placebo + gemcitabine	Locally advanced or metastatic PDAC	Completed (updated May 2025)	The primary OS endpoint was not met, underscoring the need for validated hypoxia biomarkers and target engagement measures.

ClinicalTrials.gov status was verified on July 10, 2026.

### 4.3 Multi-cargo co-delivery platforms

The tumor microenvironment is a network of interrelated obstacles. These barriers rarely function independently. Thick stromal architecture, low-oxygen areas, and immune-suppressing infiltrates often work together to promote tumor progression. When one barrier is broken, it might often make the others stronger through compensatory mechanisms. Consequently, efforts targeting a single component are frequently inadequate. Multi-cargo nanoparticle technologies provide an alternative by delivering unique therapeutic payloads to specific compartments within the tumor microenvironment, aiming for coordinated microenvironmental reprogramming rather than isolated target modifications. Several strategies enable the simultaneous delivery of multiple therapeutic agents. Core-shell designs allow the spatial separation of incompatible payloads. A polymeric core can hold hydrophobic photosensitizers or chemotherapeutic drugs, whereas a hydrophilic outer shell can hold nucleic acids or proteins. Layer-by-layer assembly offers an alternative approach to this. Researchers can sequentially deposit siRNAs, peptides, and small molecules using oppositely charged polyelectrolytes. The degradation rate of each layer can be tuned to achieve distinct kinetic release profiles. Materials that respond to stimuli provide even greater control. For example, acid-labile linkers can release a CAF-targeting drug in the acidic pericellular milieu, whereas hypoxia-sensitive azobenzene linkers can retain an oxygen-generating agent in low pO_2_ regions (77–79).

Preclinical multi-cargo systems provide insights into the potential of coordinated delivery; however, the reported combinations are better regarded as design hypotheses than as clinically validated architectures. Core-shell, layered, and stimuli-responsive carriers can keep chemically incompatible payloads apart and sequence stromal, oxygen, and immune responses. Whether that helps depends on showing that each cargo stays active, reaches its intended compartment, and is released on a biologically sensible schedule; more components, by themselves, are not evidence of synergy (27, 56, 80). Representative multi-cargo nanoparticle platforms and their intended tumor-microenvironment targets are summarized in **Table 5**.

**Table 5:** Representative multi-cargo nanoparticle platforms for integrated TME reprogramming.

Platform	Cargo combination	TME targets	Key outcome/limitation
Core-shell polymer	TGF-β-pathway modulator + checkpoint inhibitor	CAF activation + T-cell exhaustion	Potential coordinated stromal and immune modulation; preclinical and release-sequence dependent
Liposome	Catalase + STING agonist	Hypoxia relief + innate immune activation	Potential enhancement of ICD and dendritic-cell priming; requires catalytic stability and controlled innate activation
MnO_2_-loaded nanoparticle	HIF-pathway cargo + chemotherapy	Hypoxia adaptation + chemotherapy resistance	Can couple oxygen generation to drug delivery; activity depends on H_2_O_2_, pH, and tumor distribution
Layered polyelectrolyte	CAF-directed agent + myeloid-trafficking cargo	Stromal barrier + suppressive myeloid recruitment	Conceptual sequential reprogramming; manufacturing and independent release validation remain major barriers

Implications for Nanomedicine Design. Multi-cargo co-delivery depends on chemical compatibility, sequential release kinetics, and biological targeting specificity. The strongest platforms combine spatially segregated architectures with independent responsive release, allowing each payload to reach its intended microenvironmental target within the appropriate therapeutic window. Future designs should incorporate real-time monitoring to ensure that every cargo arrives at its intended destination, thereby closing the loop between sophisticated materials engineering and clinical translation.

Mammalian extracellular vesicles can shelter RNA, proteins, and small-molecule cargoes and may carry over tissue-interaction traits from their parent cells, whereas membrane-coated hybrids pair a synthetic core with selected biological interfaces. Plant-derived extracellular vesicles and exosome-like nanoparticles are potentially scalable, low-cost carriers that contain their own lipids and RNA. All of these are early stage: source heterogeneity, cargo loading, potency assays, purification, biodistribution, immunogenicity, and batch definition must be worked out before low immunogenicity or tumor tropism can be assumed (81, 82).

## 5 Limitations and translational barriers

Several barriers hinder clinical translation, and they shift sharply with the tumor context. PDAC is characterized by desmoplasia and vascular compression; glioblastoma has brain-specific barriers and infiltrative margins; breast tumors vary with molecular subtype and adipocyte-rich niches; and ovarian cancer often requires peritoneal rather than purely vascular delivery. A carrier tuned for PDAC may be useless—or worse—elsewhere. As detailed in Section 4.1, EPR heterogeneity, protein-corona remodeling, immunogenicity, and manufacturing complexity further reduce reproducibility. Real progress will require disease-specific transport models, standardized nano-biocharacterization, and prospective biomarkers, rather than a one-size-fits-all TME-targeting formulation.

For PDT-integrated platforms, the main constraints are limited and uneven illumination, photosensitizer photobleaching, oxygen depletion, and mistimed immune activation. Interstitial or endoscopic fibers and implantable micro-light sources can reach selected deep lesions, and longer-wavelength excitation extends the usable depth. Radiation- and ultrasound-triggered systems are distinct modalities and should not be considered conventional PDT. Each of these methods has its own dosimetry, device, safety, and regulatory requirements (16, 59). Studies on implantable micro-light sources illustrate the type of device engineering that spatially constrained PDT may require (59).

## 6 Future outlook

The integration of sophisticated materials science, systems biology, and clinical oncology has created new opportunities for the development of next-generation TME-targeted nanomedicine. In this section, we discuss five new trends that are likely to change the field in the next decade. We also provide our final thoughts on the main problem of turning microenvironmental reprogramming from a promising idea in the laboratory into a real-life treatment (1, 3, 83).

### 6.1 Moving toward multifunctional integration

Future TME-targeted nanoparticles may fold several microenvironmental cues into sequential or logically gated designs. One conceptual platform might briefly modulate the CAF state or matrix access, improve oxygenation, and then release an immune adjuvant only once the pre-specified conditions are met. Boolean logic delivery of this sort is still preclinical and must be judged against simpler single- and dual-function controls; the added complexity earns its place only when every sensing and release step is independently validated (29, 84).

### 6.2 Patient stratification and TME biomarkers

Patients with the same tumor type do not share the same TME architecture; therefore, applying one stromal or hypoxia strategy to an unselected population is unlikely to be effective. Useful non-invasive readouts include MRI features sensitive to matrix organization, hypoxia PET tracers, radiomic perfusion patterns, and circulating vesicle or proteomic signatures. A practical framework would assign each tumor a TME profile, including stromal density, hypoxic fraction, vascular access, and immune composition, and match that profile to a platform whose mechanism of action can be measured (5, 16, 37).

### 6.3 New technologies

Extracellular Vesicles, CRISPR Nanoparticles, and the Tumor Microbiome. Extracellular vesicles can protect complex biological cargoes and supply a biomimetic interface, and plant-derived vesicles may offer a more scalable source material; however, both require rigorous source, purity, potency, and biodistribution standards (81, 82). CRISPR-Cas cargoes carried by targeted lipid nanoparticles open a path to durable editing of immunosuppressive pathways, although off-target editing, irreversibility, and cell-specific delivery remain serious hurdles. The intratumoral microbiome may also shape immune tone and drug metabolism, especially in gastrointestinal cancers; however, microbiome-modulating nanoparticles are still being explored.

### 6.4 Artificial intelligence in nanomedicine design

Machine learning can assist nanomedicine at several levels. Formulation models can link composition, size, surface chemistry, protein corona profile, loading, and release to biological performance; mechanistic PK/PD models can simulate patient-specific dose and schedule; radiomics can estimate perfusion, hypoxia, and stromal architecture; and combined transcriptomic, proteomic, and spatial data can inform TME-based stratification. To be trustworthy, these models require curated negative and positive data, external validation, uncertainty reporting, and prospective testing; otherwise, they simply carry the bias of small preclinical datasets forward. AI should narrow the experimental search space and sharpen trial enrichment, rather than stand in for biological validation (84).

### 6.5 Regulatory and manufacturing considerations

Translating complex stimuli-responsive nanoparticles into products requires product-specific critical quality attributes and lifecycle control strategies. FDA guidance for drug products containing nanomaterials stresses the characterization of size distribution, morphology, surface properties, aggregation, composition, release, biodistribution, and the impact of manufacturing changes (85). ISO/TS 23302 provides a general framework for identifying the right measurands for nano-objects (86), while ICH quality guidelines such as Q3A and Q6A provide broad pharmaceutical guidance rather than nanomedicine-specific approval standards; therefore, they belong alongside product-specific nano-characterization, not in place of it. Scalable manufacturing should be planned early, drawing on microfluidic continuous-flow mixing, in-line size and composition monitoring, controlled polymerization, and standardized cell-free vesicle production as appropriate. Scale-up must prove that mixing history, sterilization, storage, and site transfer leave critical quality attributes, potency, and corona behavior intact (77, 79, 87).

### 6.6 Conclusion

The tumor microenvironment is more than a mere obstacle to drug delivery; it is an active and dynamic ecosystem that profoundly influences therapeutic outcomes in solid malignancies. Nanomedicine platforms that directly address this complexity by normalizing dense stroma, alleviating hypoxic adaptation, and dismantling immunosuppression represent a genuine shift from a tumor-centric perspective to one focused on the microenvironment. However, significant challenges remain, including scalable manufacturing, regulatory validation, and the need for intelligent patient stratification. Despite these hurdles, the preclinical and early clinical advancements discussed here indicate a clear trajectory in which reprogramming the tumor microenvironment will become a fundamental pillar of next-generation cancer treatment. The conversation has now evolved. The question is no longer whether nanomedicine can influence the tumor microenvironment; it is how swiftly we can translate these discoveries into meaningful clinical benefits for patients with solid tumors that remain untreatable.
